# Evaluation of Aesthetic Outcomes Following Botulinum Toxin Treatment Using Multimodal Large Language Models: A Paired Before-and-After Analysis

**DOI:** 10.1093/asjof/ojag112

**Published:** 2026-06-13

**Authors:** Ibrahim Güler, Armin Kraus, Gerrit Grieb, Henrik Stelling

## Abstract

**Background:**

Multimodal large language models (MLLMs) are increasingly applied to visual assessment tasks, yet their ability to evaluate aesthetic treatment outcomes remains unclear.

**Objectives:**

The aim of this study was to assess whether contemporary MLLMs can identify treatment state and detect region-specific aesthetic improvement following botulinum neurotoxin treatment.

**Methods:**

In this observational study, 23 paired facial image cases (46 images; 460 model evaluations) were analyzed. Four MLLMs (GPT-5.4 Pro [OpenAI, San Francisco, CA], Grok 4.1 [xAI, San Francisco, CA], Gemini 3.1 Pro [Google DeepMind, Mountain View, CA], and Claude Opus 4.6 [Anthropic, San Francisco, CA]) performed 5 independent inference runs per case. Models identified the posttreatment image and assessed regional improvement (forehead, glabella, and periorbital). Accuracy, sensitivity, specificity, balanced accuracy, Matthews correlation coefficient, and Fleiss’ *κ* were calculated descriptively. Performance was compared with majority-class baselines. Exploratory outputs included aesthetic scores and apparent age estimates.

**Results:**

All models identified the posttreatment image (100% accuracy). Region-specific improvement detection frequently failed to exceed majority-class baselines (65.2%-91.3%). Gemini 3.1 Pro showed the highest performance for the forehead (74.8%) and glabella (63.5%), whereas no model reached the periorbital baseline. Inter-run reliability varied widely (*κ* −0.113 to 0.719). High reliability did not imply correctness. All models systematically overestimated improvement (62.6%-94.8% of predictions exceeded ground truth). False positives exceeded false negatives in all 12 model–task combinations. Exploratory outputs indicated perceived rejuvenation.

**Conclusions:**

MLLMs recognize the format of aesthetic change but not its clinical nuance. Bridging this gap requires more than improved accuracy: a coordinated agenda of domain-specific fine-tuning, expert-rater benchmarking, and structured outcome frameworks, supported by governance of training-data provenance and clear safeguards before any clinical or research deployment.

**Level of Evidence: 5 (Therapeutic):**

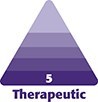

Botulinum neurotoxin (BoNT) injections are among the most frequently performed procedures in aesthetic medicine worldwide, with established indications for the treatment of dynamic wrinkles in the forehead, glabella, and periorbital regions.^1-3^ Treatment planning and outcome assessment rely on visual evaluation of wrinkle presence, severity, and regional distribution, a process that remains inherently subjective despite the development of structured grading systems.^4-6^

The subjective nature of treatment outcome evaluation presents challenges for clinical practice and research. Inter-rater variability in wrinkle severity grading is well documented, and even experienced clinicians may disagree on the degree of treatment response when evaluating standardized photographs.^4^ This subjectivity limits the comparability of outcome data across studies and clinical settings, underscoring the need for complementary, observer-independent assessment tools.

Recent advances in artificial intelligence (AI), particularly since the publication of Attention Is All You Need by Vaswani and colleagues in 2017, have driven the rapid evolution of large language models and, more recently, multimodal large language models (MLLMs) capable of processing both text and image inputs simultaneously.^7-9^ These models have shown variable performance across medical image interpretation tasks, including radiographic fracture detection and dermatological assessment.^10-13^ In a recent pilot study, Saeed et al demonstrated near-perfect agreement between a contemporary MLLM and board-certified plastic surgeons in facelift candidacy assessment from standardized facial photographs, suggesting that such models can approximate expert-level visual decision making under controlled conditions.^14^ In parallel, deep learning–based approaches have been used to objectively quantify changes in facial expressions following BoNT-A injections.^15^ As facial expressions are central to emotional communication, such alterations may also influence social perception. Consistently, upper-face treatment has been shown to modulate how facial emotions are attributed and perceived by observers.^16^

Most evaluations of MLLMs in facial aesthetics have employed single-image inference, in which models analyze 1 image at a time without access to a comparative reference state.^14,17^ In contrast to clinical practice, where outcomes are typically assessed by directly comparing paired pre- and posttreatment photographs, it remains unclear whether MLLMs can perform this comparative visual task and accurately identify region-specific aesthetic changes.

More broadly, MLLM evaluations across medical domains have typically reported accuracy from a single inference run per case, without assessing the consistency of model outputs across repeated evaluations.^10-14^ Given the stochastic nature of MLLM inference, a single run may not reliably represent model behavior. The use of multiple independent runs allows assessment of inter-run reliability, which has been identified as a critical and frequently overlooked dimension of MLLM performance evaluation.^18-20^

BoNT outcome assessment relies on visual evaluation of region-specific wrinkle changes and is subject to known inter-rater variability, highlighting the need for complementary, observer-independent assessment approaches. The ability to distinguish pre- from posttreatment images may not imply the capacity to assess the nature or extent of aesthetic change at a regional level. The present study addresses these gaps by evaluating whether 4 contemporary MLLMs can assess aesthetic treatment outcomes from paired before-and-after facial images following BoNT treatment of the upper face. Models were tasked with identifying the posttreatment image, detecting region-specific improvement in 3 clinically relevant areas (forehead, glabella, and periorbital), and generating exploratory aesthetic scores and age estimates. Each case was evaluated across 5 independent runs to assess both classification performance and inter-run reliability.

## METHODS

### Study Design and Objectives

This observational study evaluated the performance of 4 contemporary MLLMs in assessing aesthetic treatment outcomes from paired before-and-after facial images following BoNT treatment. The study employed a paired-image comparison design in which each model simultaneously viewed both the pretreatment and posttreatment image and was tasked with identifying the posttreatment image and evaluating regional aesthetic changes.

The study had 3 primary objectives and 1 exploratory objective:

1. to assess whether models can correctly identify the posttreatment image in paired comparisons;
2. to quantify performance of region-specific aesthetic improvement detection, including accuracy, sensitivity, specificity, and comparison to majority-class baselines;
3. to evaluate inter-run reliability of model outputs for categorical classification tasks using Fleiss’ *κ*^21^;
4. to characterize exploratory model-generated aesthetic change scores and apparent age estimates as descriptive endpoints without ground-truth validation.

All analyses were descriptive. No inferential statistical testing was performed because of the fixed dataset, lack of a sampling framework, and repeated-measures structure.

### Dataset and Image Preparation

A total of 23 paired facial image cases were derived from a publicly available facial image dataset (CC BY-NC-ND 4.0 license). The dataset does not include structured metadata on patient age, anatomical treatment region, injection dose, product brand, or time interval between treatment and posttreatment photography.^22^ The source dataset comprised 23 paired cases, all of which were included without exclusion or selective sampling. Sample size was therefore determined by the dataset boundary, and no formal a priori sample size calculation was performed, consistent with the descriptive observational design of the study.

Each case consisted of 1 pretreatment and 1 posttreatment frontal portrait of the same individual following BoNT treatment of the upper face (forehead, glabella, and periorbital regions). Images comprised a mix of neutral and dynamic facial expressions. Within each paired case, position and cropping were generally comparable, allowing meaningful before-and-after comparison. Across cases, however, framing, distance, and head positioning varied. Lighting and acquisition conditions were not standardized, reflecting the heterogeneity of the source dataset. Skin types were visually assessed as Fitzpatrick I to III.

For each case, the pretreatment and posttreatment images were uploaded as 2 separate image files within the same prompt session, without identifying filenames, metadata, or side-by-side compositional merging. The 2 images were presented to the model simultaneously as an unordered pair, without spatial positional cues linking image identity to treatment state and without a fixed upload order across cases or runs.

Wrinkle presence before-and-after treatment was assessed for each region by consensus among 2 board-certified plastic surgeons, 1 plastic surgery resident, and 1 physician with regular clinical experience in BoNT administration. Reviewers were not blinded to treatment state, because the assessment of regional improvement inherently required paired evaluation of both images. Each case was discussed independently before reaching consensus; individual preconsensus ratings were not formally recorded in a structured format. Wrinkle presence was assessed independently for each image per region (binary: 0 = absent, 1 = present). Regional improvement was derived as any visible change from the pretreatment to the posttreatment image, including reduction in severity or complete resolution of wrinkles. The number of improved regions per case, derived from region-specific annotations, served as the reference standard for overall improvement.

### Models Under Evaluation

Four contemporary MLLMs representative of current state-of-the-art systems were evaluated:

- GPT-5.4 Pro (OpenAI, San Francisco, CA)
- Grok 4.1 (xAI, San Francisco, CA)
- Gemini 3.1 Pro (Google DeepMind, Mountain View, CA)
- Claude Opus 4.6 (Anthropic, San Francisco, CA)

All models were accessed through their official web interfaces rather than through an application programming interface (API), and no parameter adjustments (eg, temperature or top-p) were applied. This approach was chosen to approximate how these systems are typically used in clinical and research settings. Model versions correspond to the publicly available system state at the time of data collection in March 2026.

### Prompting Strategy and Inference Protocol

Each model underwent 5 independent inference runs per case using an identical, standardized zero-shot prompt. Specifically, models were asked to determine which of the 2 images corresponded to the posttreatment state and to assess aesthetic improvement in 3 clinically relevant regions of the upper face (forehead, glabella, and periorbital areas; binary: 0 = no improvement, 1 = improvement), report the total number of improved regions (integer, 0-3), assign an overall aesthetic change score reflecting perceived visible improvement (integer, 0-10; 0 = no visible improvement, 10 = maximal visible improvement), and estimate the apparent age before-and-after treatment.

This structured prompt was designed to reflect key elements of routine clinical assessment in aesthetic practice, where treatment-state recognition, regional changes, and overall aesthetic outcomes are evaluated in parallel.

To reduce carryover between runs, each inference was conducted in a fresh browser session with cleared cache and cookies, without previous conversational context, and with chat history and data-sharing or training settings disabled where applicable. No conversational memory, feedback, or adaptive prompting was permitted. The prompt is provided in the Appendix.

### Outcome Measures

The following outcome measures were evaluated in relation to the study objectives:

1. identification accuracy of the posttreatment image in paired comparisons;
2. performance of region-specific improvement detection, including accuracy, sensitivity, and specificity for the forehead, glabella, and periorbital regions;
3. accuracy for predicting the total number of improved regions;
4. evaluation of inter-run reliability of categorical model outputs using Fleiss’ *κ*.

Exploratory outcomes included:

1. aesthetic change scores, in which models rated overall visible improvement on an unvalidated numerical scale from 0 (no visible improvement) to 10 (maximal visible improvement), analyzed descriptively and stratified by the ground truth number of improved regions;
2. estimated apparent age before-and-after treatment, with the perceived age difference (Δ age = estimated age before minus estimated age after) reported as a measure of perceived rejuvenation.

These outcomes were exploratory and descriptive in nature, lacking external ground truth, and should be interpreted with caution as model-generated outputs rather than validated measurements.

### Statistical Analysis

All analyses were performed using Python 3.12 (Python Software Foundation, Wilmington, DE) with standard statistical and data visualization libraries. Classification accuracy was computed per model per run as the number of correct classifications divided by the total number of cases (*n* = 23) and summarized as mean ± standard deviation (SD) across 5 runs with the range (minimum to maximum). Sensitivity and specificity were computed per model per run per task, with the positive class defined as the presence of improvement (coded as 1) for regional detection tasks. Sensitivity and specificity values reflect discrete case-level outcomes and may exhibit limited granularity because of small sample sizes.

Inter-run reliability was assessed using Fleiss’ *κ*, computed on raw model outputs for categorical classification tasks rather than on correct/incorrect classifications, reflecting consistency of model responses across runs rather than agreement with ground truth.^21^ In cases of invariant outputs, Fleiss’ *κ* may be inflated or nonestimable and should be interpreted with caution, as this may reflect limited variability in model responses. *κ* values were interpreted according to Landis and Koch thresholds: <0.20 slight, 0.21 to 0.40 fair, 0.41 to 0.60 moderate, 0.61 to 0.80 substantial, and 0.81 to 1.00 almost perfect agreement.^23^

The majority-class baseline was defined as the accuracy achieved by always predicting the most frequent ground-truth category for each task; performance at or below this threshold indicates no incremental information beyond the trivial constant predictor.

Balanced accuracy, defined as the mean of sensitivity and specificity, and the Matthews correlation coefficient (MCC) were additionally computed for binary classification tasks to account for substantial class imbalance, particularly in the periorbital task. Both metrics were calculated per run and aggregated as mean ± SD across runs. Aggregate confusion matrix counts (true positives, true negatives, false positives, and false negatives) summed across all 5 runs per model per task are presented in the Results.

Overestimation of aesthetic improvement was assessed by comparing the model-predicted number of improved regions to the ground-truth value for each case and run. Predictions were classified as overestimation (predicted greater than ground truth), exact match, or underestimation (predicted less than ground truth), and the proportion of each category was reported per model.

Exploratory variables (aesthetic change scores and age estimates) were reported descriptively as mean ± SD.

### Ethical Considerations

Ethical review and approval were not required for this study, as confirmed by the responsible institutional ethics committee (reference number 25-499-ANF, December 15, 2025). The study was based exclusively on publicly available, fully anonymized data without any interaction with human participants.

## RESULTS

### Dataset Characteristics

The dataset comprised 23 paired facial image cases, each consisting of pre- and posttreatment photographs presented together. Each case was evaluated by 4 MLLMs across 5 independent runs, yielding 460 model evaluations per task (23 cases × 4 models × 5 runs). Ground-truth annotations confirmed forehead improvement in 15 cases (65.2%), glabella improvement in 9 cases (39.1%), and periorbital improvement in 2 cases (8.7%). The total number of improved regions ranged from 1 to 2 (Table 1). The ground-truth distribution showed substantial class imbalance (Table 1). No ground truth was available for aesthetic change scores or age estimates, which were treated as exploratory model-generated outputs.

**Table 1.** Dataset Characteristics and Ground-Truth Distribution

Variable	Category	*n*	%
Total paired cases		23	
Forehead improvement (GT)	Improved	15	65.2
	Not improved	8	34.8
Glabella improvement (GT)	Improved	9	39.1
	Not improved	14	60.9
Periorbital improvement (GT)	Improved	2	8.7
	Not improved	21	91.3
Number of improved regions (GT)	1 region	20	87.0
	2 regions	3	13.0

Ground-truth classifications were established by consensus among the study authors (2 board-certified plastic surgeons, 1 plastic surgery resident, and 1 physician with regular clinical experience in BoNT administration).

GT, ground truth.

All models achieved 100% accuracy in identifying the posttreatment image across all runs. This result reflects performance within the paired comparison setting and does not constitute an independent or generalizable performance measure.

### Region-Specific Improvement Detection

**Figure 1.**
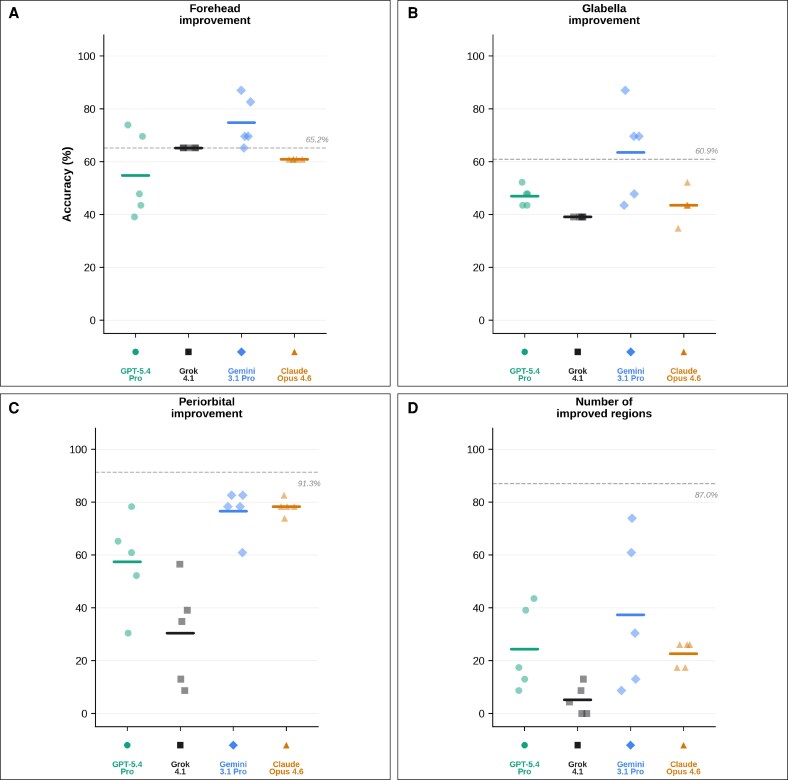
Model performance across tasks: accuracy relative to majority-class baselines in paired aesthetic assessment. Model performance across 4 classification tasks in paired before-and-after facial image assessment. Panels show per-run accuracy (5 independent runs per model) for (A) forehead improvement, (B) glabella improvement, (C) periorbital improvement, and (D) number of improved regions. Each point represents 1 run per model; horizontal bars indicate mean accuracy across runs. Dashed horizontal lines denote the majority-class baseline for each task (forehead: 65.2%; glabella: 60.9%; periorbital: 91.3%; number of improved regions: 87.0%), defined as the accuracy achieved by always predicting the most frequent ground-truth category.

Performance for region-specific improvement detection varied across models and facial regions (Table 2; Figure 1). Performance frequently failed to exceed majority-class baselines across regions.

**Table 2.** Classification Performance Across Tasks and Models

Task	Model	Accuracy (mean ± SD)	Range	Sensitivity (mean ± SD)	Specificity (mean ± SD)	Balanced acc. (mean ± SD)	MCC (mean ± SD)	Fleiss’ *κ*	Majority baseline
Forehead improvement	GPT-5.4 Pro	54.8 ± 15.9	39.1-73.9	74.7 ± 22.3	17.5 ± 14.3	46.1 ± 13.9	−0.043 ± 0.351	0.180	65.2%
	Grok 4.1	65.2 ± 0.0	65.2-65.2	100.0 ± 0.0	0.0 ± 0.0	50.0 ± 0.0	n.d.^a^	—^a^	
	Gemini 3.1 Pro	74.8 ± 9.4	65.2-87.0	100.0 ± 0.0	27.5 ± 27.1	63.8 ± 13.5	0.483 ± 0.224	0.146	
	Claude Opus 4.6	60.9 ± 0.0	60.9-60.9	93.3 ± 0.0	0.0 ± 0.0	46.7 ± 0.0	−0.156 ± 0.000	1.000^b^	
Glabella improvement	GPT-5.4 Pro	47.0 ± 3.6	43.5-52.2	100.0 ± 0.0	12.9 ± 6.0	56.4 ± 3.0	0.229 ± 0.059	0.458	60.9%
	Grok 4.1	39.1 ± 0.0	39.1-39.1	97.8 ± 5.0	1.4 ± 3.2	49.6 ± 0.9	−0.069^c^	−0.018	
	Gemini 3.1 Pro	63.5 ± 17.8	43.5-87.0	97.8 ± 5.0	41.4 ± 30.1	69.6 ± 14.5	0.435 ± 0.237	0.346	
	Claude Opus 4.6	43.5 ± 6.1	34.8-52.2	88.9 ± 0.0	14.3 ± 10.1	51.6 ± 5.1	0.016 ± 0.172	0.617	
Periorbital improvement	GPT-5.4 Pro	57.4 ± 17.8	30.4-78.3	90.0 ± 22.4	54.3 ± 20.9	72.1 ± 8.2	0.264 ± 0.078	0.217	91.3%
	Grok 4.1	30.4 ± 19.7	8.7-56.5	90.0 ± 22.4	24.8 ± 21.9	57.4 ± 13.8	0.117 ± 0.172	−0.113	
	Gemini 3.1 Pro	76.5 ± 9.0	60.9-82.6	70.0 ± 27.4	77.1 ± 11.9	73.6 ± 9.6	0.306 ± 0.098	0.426	
	Claude Opus 4.6	78.3 ± 3.1	73.9-82.6	50.0 ± 35.4	81.0 ± 3.4	65.5 ± 16.9	0.203 ± 0.219	0.719	
Number of improved regions	GPT-5.4 Pro	24.3 ± 15.9	8.7-43.5	—	—	—	—	0.148	87.0%
	Grok 4.1	5.2 ± 5.7	0.0-13.0	—	—	—	—	−0.107	
	Gemini 3.1 Pro	37.4 ± 28.9	8.7-73.9	—	—	—	—	0.181	
	Claude Opus 4.6	22.6 ± 4.8	17.4-26.1	—	—	—	—	0.583	

Values are expressed as percentages for accuracy, sensitivity, specificity, and balanced accuracy. MCC, Matthews correlation coefficient (range −1 to +1). Fleiss’ *κ* computed on raw model outputs across 5 runs. Balanced accuracy was computed as the mean of sensitivity and specificity per run; MCC was computed using the standard definition. Both were aggregated as mean ± SD across runs. SD, standard deviation.

^a^Not estimable/not determinable because of invariant model output (identical response across cases or runs).

^b^Trivial perfect agreement because of identical response patterns across runs.

^c^Based on 1 of 5 noninvariant runs. Sensitivity, specificity, balanced accuracy, and MCC are not reported for the multiclass task (number of improved regions).

#### Forehead Improvement (Majority-Class Baseline: 65.2%)

Gemini 3.1 Pro had the highest mean accuracy (74.8 ± 9.4%) and exceeded or matched the baseline across runs. Grok 4.1 matched the baseline exactly (65.2 ± 0.0%) with invariant output across all runs and cases (all classified as improved; Fleiss’ *κ* not estimable). Claude Opus 4.6 showed a consistent response pattern across runs (60.9 ± 0.0%, *κ* = 1.000, reflecting invariant or near-invariant outputs across runs rather than necessarily meaningful agreement) but fell below the baseline. GPT-5.4 Pro showed the widest inter-run variability (54.8 ± 15.9%; range, 39.1%-73.9%; *κ* = 0.180).

#### Glabella Improvement (Majority-Class Baseline: 60.9%)

Gemini 3.1 Pro had the highest mean accuracy (63.5 ± 17.8%) but with wide inter-run variability (range, 43.5%-87.0%, *κ* = 0.346). GPT-5.4 Pro (47.0 ± 3.6%, *κ* = 0.458), Claude Opus 4.6 (43.5 ± 6.1%, *κ* = 0.617), and Grok 4.1 (39.1 ± 0.0%, *κ* = −0.018) did not exceed the baseline.

#### Periorbital Improvement (Majority-Class Baseline: 91.3%)

No model reached this threshold. Claude Opus 4.6 (78.3 ± 3.1%, *κ* = 0.719) and Gemini 3.1 Pro (76.5 ± 9.0%, *κ* = 0.426) showed the highest accuracy, followed by GPT-5.4 Pro (57.4 ± 17.8%, *κ* = 0.217) and Grok 4.1 (30.4 ± 19.7%, *κ* = −0.113). Sensitivity estimates were unstable because of the small number of positive cases (*n* = 2), ranging from 0.0% to 100.0% within individual models across runs.

Across all 3 regions, a consistent pattern emerged: high sensitivity (range, 50.0%-100.0%) combined with low specificity (range, 0.0%-81.0%), indicating systematic overcalling of improvement.

Balanced accuracy, which accounts for class imbalance by averaging sensitivity and specificity, confirmed the pattern observed with raw accuracy: values ranged from 46.1% to 63.8% for forehead improvement, 49.6% to 69.6% for glabella improvement, and 57.4% to 73.6% for periorbital improvement (Table 2). MCC values were weak to moderate across most model–task combinations (range, −0.156 to 0.483 for forehead, −0.069 to 0.435 for glabella, and 0.117 to 0.306 for periorbital), with several values near or below zero indicating performance at or below chance level.

### Number of Improved Regions and Overestimation of Aesthetic Improvement

**Figure 2.**
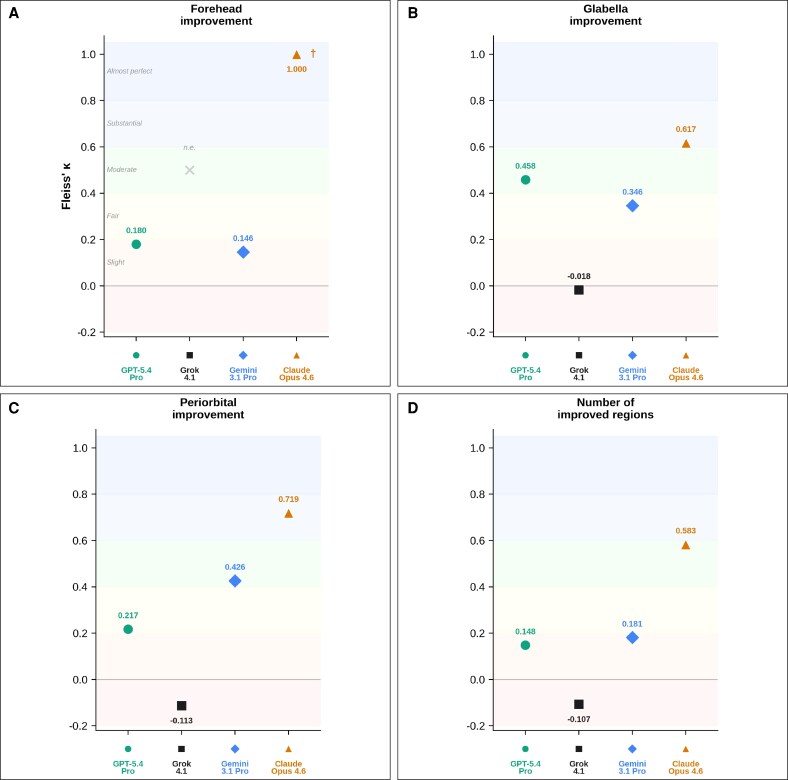
Inter-run reliability of model outputs across 4 classification tasks, quantified using Fleiss’ *κ*. Panels show *κ* values for (A) forehead improvement, (B) glabella improvement, (C) periorbital improvement, and (D) number of improved regions. Each point represents the agreement across the 5 independent runs for a given model and task. Background shading indicates agreement categories according to Landis and Koch (slight, fair, moderate, substantial, and almost perfect). Negative *κ* values indicate agreement below chance. For models producing identical response patterns across all 5 runs, *κ* = 1.000 reflects perfect run-to-run consistency regardless of accuracy (†, eg, Claude in A). Where a model produced the same response for all cases across all runs, *κ* was not estimable (n.e., eg, Grok in A).

Accuracy for predicting the number of improved regions was low across all models and consistently far below the majority-class baseline of 87.0%: Gemini 3.1 Pro 37.4 ± 28.9%, GPT-5.4 Pro 24.3 ± 15.9%, Claude Opus 4.6 22.6 ± 4.8%, and Grok 4.1 5.2 ± 5.7% (Table 2). Inter-run reliability ranged from moderate (Claude Opus 4.6, *κ* = 0.583) to negative (Grok 4.1, *κ* = −0.107; Figure 2). This pattern is consistent with overestimation of treatment effects.

Models systematically overestimated aesthetic improvement across regions and cases (Table 3). Across all models, 62.6% to 94.8% of predictions for the number of improved regions exceeded the ground-truth value, whereas underestimation occurred in 0.0% to 1.7% of evaluations. The mean overestimation ranged from +0.78 regions (Gemini 3.1 Pro) to +1.62 regions (Grok 4.1). Grok 4.1 predicted 3 improved regions in 74.8% of all evaluations (86/115), whereas the ground-truth maximum was 2 improved regions.

**Table 3.** Estimation of Improved Regions

Model	Overestimation	Exact match	Underestimation	Mean difference
GPT-5.4 Pro	85/115 (73.9%)	28/115 (24.3%)	2/115 (1.7%)	+1.06
Grok 4.1	109/115 (94.8%)	6/115 (5.2%)	0/115 (0.0%)	+1.62
Gemini 3.1 Pro	72/115 (62.6%)	43/115 (37.4%)	0/115 (0.0%)	+0.78
Claude Opus 4.6	89/115 (77.4%)	26/115 (22.6%)	0/115 (0.0%)	+0.89

Mean difference = mean (predicted minus ground truth) number of improved regions across all 115 evaluations (23 cases × 5 runs) per model. Positive values indicate systematic overestimation.

Perfect identification of treatment state did not translate into accurate assessment of aesthetic improvement. Models frequently failed to outperform simple baseline strategies for regional improvement detection and consistently overstated the extent of visible changes.

Aggregate confusion matrix counts across all 5 runs confirmed a pronounced asymmetry between false positives and false negatives across tasks and models (Table 4). False positives exceeded false negatives in all 12 model–task combinations, with ratios ranging from 4:1 to 79:1 for glabella and periorbital improvement, reflecting the systematic directional bias toward overcalling improvement.

**Table 4.** Aggregate Confusion Matrix Counts per Model and Task

Task	Model	TP	TN	FP	FN	Total
Forehead	GPT-5.4 Pro	56	7	33	19	115
	Grok 4.1	75	0	40	0	115
	Gemini 3.1 Pro	75	11	29	0	115
	Claude Opus 4.6	70	0	40	5	115
Glabella	GPT-5.4 Pro	45	9	61	0	115
	Grok 4.1	44	1	69	1	115
	Gemini 3.1 Pro	44	29	41	1	115
	Claude Opus 4.6	40	10	60	5	115
Periorbital	GPT-5.4 Pro	9	57	48	1	115
	Grok 4.1	9	26	79	1	115
	Gemini 3.1 Pro	7	81	24	3	115
	Claude Opus 4.6	5	85	20	5	115

Counts aggregated across all 5 inference runs per case (23 cases × 5 runs = 115 evaluations per cell). A positive class defined as the presence of improvement.

FN, false negative; FP, false positive; TN, true negative; TP, true positive.

### Exploratory Aesthetic Change Scores

Model-generated aesthetic change scores (scale 0-10) are summarized in Table 5. Mean scores across all cases and runs ranged from 5.43 ± 1.24 (Claude Opus 4.6) to 6.86 ± 1.03 (Gemini 3.1 Pro). When stratified by the ground truth number of improved regions, cases with 2 improved regions received marginally higher aesthetic scores than cases with 1 improved region for 3 of 4 models, although differences were small.

**Table 5.** Exploratory Aesthetic Change Scores and Age Estimates

Variable	Model	Overall mean ± SD	GT 1 region (*n* = 20)	GT 2 regions (*n* = 3)
Aesthetic change score (0-10)	GPT-5.4 Pro	5.44 ± 1.43	5.40 ± 1.48	5.73 ± 1.03
	Grok 4.1	6.42 ± 1.15	6.47 ± 1.16	6.07 ± 1.10
	Gemini 3.1 Pro	6.86 ± 1.03	6.78 ± 1.02	7.40 ± 0.91
	Claude Opus 4.6	5.43 ± 1.24	5.34 ± 1.29	6.07 ± 0.46
Age before (years)	GPT-5.4 Pro	39.6 ± 9.9		
	Grok 4.1	43.7 ± 9.9		
	Gemini 3.1 Pro	40.9 ± 9.8		
	Claude Opus 4.6	38.9 ± 9.8		
Age after (years)	GPT-5.4 Pro	36.7 ± 9.0		
	Grok 4.1	40.1 ± 9.1		
	Gemini 3.1 Pro	36.7 ± 8.7		
	Claude Opus 4.6	35.2 ± 8.8		
Δ Age (years)	GPT-5.4 Pro	2.95 ± 1.23		
	Grok 4.1	3.60 ± 1.33		
	Gemini 3.1 Pro	4.19 ± 1.71		
	Claude Opus 4.6	3.66 ± 1.69		

Aesthetic change scores and age estimates are reported as mean ± SD across all evaluations (23 cases ×5 runs = 115 per model). Aesthetic scores stratified by GT-improved regions represent mean across all runs for cases within each GT category. Δ age = estimated age before minus estimated age after; positive values indicate perceived younger appearance after treatment.

GT, ground truth; SD, standard deviation.

### Exploratory Age Estimates

All 4 models consistently estimated lower apparent age for the posttreatment side compared with the pretreatment side (Table 5). The mean perceived age difference (Δ age) ranged from 2.95 ± 1.23 years (GPT-5.4 Pro) to 4.19 ± 1.71 years (Gemini 3.1 Pro). Across all 460 individual evaluations, no model estimated the posttreatment image to appear older than the pretreatment image.

## DISCUSSION

The central finding of this study is a striking dissociation between treatment-state recognition and clinical outcome assessment. All 4 models identified the posttreatment image with 100% accuracy across all runs yet consistently failed to detect region-specific aesthetic changes and systematically overestimated the extent of improvement. This pattern suggests that current MLLMs may distinguish global visual differences between paired images without reliably identifying localized clinically relevant changes.

The perfect performance on treatment-side identification is consistent with exposure to large amounts of curated before-and-after facial imagery during model training. Such photographs are widely shared across social media platforms, patient testimonial websites, and marketing materials, creating a highly patterned corpus of paired images in which the posttreatment state is almost invariably presented as the more favorable image. Models may therefore rely on global visual cues, such as smoother skin texture or reduced shadowing, to classify treatment state, rather than performing the localized anatomical reasoning required for region-specific evaluation. This behavior is consistent with shortcut learning, in which models exploit superficial statistical regularities rather than task-relevant features, a phenomenon described as a general limitation of deep neural networks.^24^ This interpretation aligns with previous work highlighting that AI systems in plastic surgery are subject to bias, variability, and limitations related to training data, model design, and application context.^25,26^

Once the task required more than binary classification, performance deteriorated in a manner that followed a clear complexity gradient. For the simplest clinical task, forehead improvement detection (majority-class baseline 65.2%), 1 model exceeded the baseline across runs. For glabella improvement (baseline 60.9%), only 1 model marginally surpassed the threshold. For periorbital improvement (baseline 91.3%), no model approached the baseline. The number of improved regions, which requires integrating assessments across all 3 regions, yielded the lowest accuracies (5.2%-37.4% vs a baseline of 87.0%). This progressive decline indicates that model performance degrades as tasks demand finer spatial discrimination and multiregion synthesis.

Several factors may contribute to region-specific differences. Training data distribution shapes model behavior: publicly available before-and-after imagery is not uniform across facial regions and tends to emphasize prominent changes. From a computer vision perspective, larger- and higher-contrast features are easier to detect than fine-grained, low-contrast structures, and the patch-based processing of current vision encoders may further favor dominant patterns over subtle localized changes. Similar to human observers, whose assessments are shaped by training and experience, model performance reflects learned biases in the underlying data.

Systematic overestimation of treatment effects emerged as a dominant pattern across all models and tasks. Between 62.6% and 94.8% of predictions for the number of improved regions exceeded the ground-truth value, whereas underestimation was virtually absent (0.0%-1.7%). This directional bias is reflected in the sensitivity–specificity asymmetry observed across regions: sensitivity ranged from 50.0% to 100.0%, whereas specificity ranged from 0.0% to 81.0%, indicating that models overwhelmingly defaulted to predicting improvement regardless of the actual clinical state. One possible explanation is that training corpora disproportionately contain examples in which treatment is associated with visible improvement, reinforcing a learned prior toward positive outcomes. In clinical terms, this means that current MLLMs are more likely to confirm improvement than to identify its absence, a tendency that could lead to overoptimistic outcome reporting if such tools were used without expert oversight.

These patterns are corroborated by the additional class-imbalance-robust metrics: balanced accuracy values clustered between 46.1% and 73.6% across regions, and MCC values remained weak across most model–task combinations (range, −0.156 to 0.483), indicating that observed accuracy largely reflected the dominant majority class rather than meaningful classification ability. Aggregate confusion matrix analysis (Table 4) confirmed this directional bias, with false positives exceeding false negatives in all 12 model–task combinations.

Inter-run reliability, assessed using Fleiss’ *κ*, varied widely across models and tasks (*κ* from −0.113 to 0.719). Two observations merit particular attention. High reliability did not imply correctness. One model achieved *κ* = 1.000 for forehead improvement but fell below the majority-class baseline, reflecting a consistent but incorrect response pattern. Conversely, a different model produced invariant outputs across all cases and runs for the same task, rendering *κ* not estimable. These findings illustrate that inter-run reliability and classification accuracy capture fundamentally different dimensions of model behavior. Consistency of output should not be conflated with validity of output, an important distinction for any framework that seeks to benchmark MLLM performance in clinical contexts.^18-20^

Exploratory aesthetic change scores and age estimates, while lacking external ground truth and therefore not amenable to accuracy evaluation, revealed additional patterns consistent with a positivity bias. Mean aesthetic change scores ranged from 5.43 to 6.86 on a 0-to-10 scale, and all 460 individual age estimates indicated perceived rejuvenation following treatment, with no single evaluation estimating the posttreatment image to appear older. Previous work using convolutional neural networks for facial age estimation demonstrated that AI-derived reductions in apparent age after facelift surgery are positively correlated with patient-reported satisfaction.^27^ In the present study, however, the consistency of perceived rejuvenation across all cases, including those with minimal documented change, suggests that age-related outputs may similarly reflect learned associations between treatment and positive outcomes rather than case-specific visual assessment.

These findings should be interpreted in the context of the growing body of literature evaluating AI and MLLMs in aesthetic surgery. Early evaluations of MLLMs in aesthetic surgery have demonstrated variable and often suboptimal performance across key text-based clinical tasks, including patient education, postoperative triage, and management of patient-reported concerns.^25,26,28-30^ The present study extends this work to visual outcome assessment, a domain in which MLLMs are increasingly expected to contribute given their multimodal capabilities. A recent comprehensive review identified clinical outcome assessment as one of several application domains of AI in plastic surgery, while highlighting substantial heterogeneity in study design and outcome metrics, and noting that subjective outcome assessment may be particularly prone to bias.^26^ Our findings confirm this concern: models performed well on the most superficial visual task but failed precisely where clinical judgment requires nuance.

The present study deliberately evaluated general-purpose MLLMs rather than task-specific deep learning systems. Contemporary MLLMs incorporate state-of-the-art vision transformer architectures trained at unprecedented scale and are readily accessible to clinicians, researchers, and patients through standard web interfaces without specialized hardware or machine learning expertise. Their performance therefore provides a clinically meaningful baseline that any future domain-specific model would need to exceed. Dedicated systems trained on expert-annotated datasets may achieve higher task-specific accuracy, but their accessibility and deployment pathway differ fundamentally.

The clinical implications are direct. Current general-purpose MLLMs are not suitable for independent aesthetic outcome assessment. Their systematic bias toward overestimating improvement, combined with poor specificity and inconsistent reliability, means that model-generated evaluations could overstate treatment effects, inflate patient expectations, and produce misleading data in research contexts. These risks underscore the need for expert oversight and task-specific validation before any clinical or research deployment.

This is particularly relevant in light of ongoing efforts to formalize aesthetic outcome assessment through structured frameworks and composite indices, which may represent a necessary foundation for the future integration of AI-assisted evaluation tools in clinical research.^31^ Should these tools be deployed prematurely, the positivity bias documented here could systematically inflate reported efficacy.

A further consideration arises from regulatory and content availability differences across domains. In Germany, the use of before-and-after photographs to advertise nonmedically indicated aesthetic procedures is restricted under advertising regulations for medicinal products, and a 2025 ruling by Germany's Federal Court of Justice confirmed that this prohibition extends to minimally invasive aesthetic procedures, including BoNT injections.^32^ Internationally, however, such imagery remains widely disseminated across digital platforms.

If the observed model behavior is indeed influenced by exposure to large amounts of curated before-and-after imagery, this would imply that performance may vary across domains depending on the availability of such visual data in the training corpus. In areas where publicly accessible imagery is more limited or restricted, such as genital aesthetic procedures, model performance might be expected to differ. This hypothesis remains speculative and would require targeted investigation in future studies.

### Limitations

Several limitations should be acknowledged. The dataset comprised only 23 paired cases from a single publicly available source, limiting generalizability.^22^ However, the 5-run design yielded 460 model evaluations per task, providing a more comprehensive assessment of model behavior than single-run approaches.^19^ Given the small sample size, pronounced class imbalance, and nonindependence of repeated model evaluations, performance metrics (including sensitivity, specificity, and Fleiss’ *κ*) are inherently unstable and should be interpreted with caution. The analyses are therefore descriptive and intended to characterize observed model behavior rather than to provide definitive estimates of diagnostic performance.

The dataset showed limited image standardization, with mixed neutral and dynamic facial expressions, variable lighting, and nonstandardized pose and was predominantly composed of individuals with Fitzpatrick skin types I to III. These factors may introduce variability beyond clinical changes and limit the generalizability of the findings to populations with darker skin types (Fitzpatrick IV-VI). In addition, the source dataset did not include structured metadata on patient age, anatomical treatment region, injection dose, product brand, or time interval between treatment and posttreatment photography, precluding stratified subgroup analyses on these variables. All cases were sourced from a dataset explicitly labeled as BoNT treatment cases and showed clear posttreatment effects of upper facial BoNT administration. However, concurrent or previous non-BoNT procedures (eg, dermal fillers) could not be excluded based on the available data and may have introduced visual changes unrelated to BoNT that influenced model assessments.^22^

Ground truth was established by expert consensus using a binary classification (wrinkle presence vs absence), chosen for clinical pragmatism and ground-truth tractability. This binary operationalization does not capture the full spectrum of severity gradation and may underestimate model performance in cases of partial improvement.^3-6^

Preconsensus inter-rater agreement was not formally quantified, and individual ratings were not retained for retrospective computation; prospective documentation of inter-rater agreement is a defined improvement for future studies.

The paired presentation format, in which models viewed pretreatment and posttreatment images simultaneously, may overestimate real-world performance compared with single-image settings in which no reference state is available.

All models were accessed through their official web interfaces without adjustment of API-level parameters such as temperature or top-p. Although this approach reflects typical real-world usage in clinical and research contexts, it is possible that further performance gains could have been achieved through systematic parameter optimization.

Prompt structure has been shown to substantially influence MLLM outputs.^33-35^ The present results reflect performance under a single, fixed zero-shot prompt without chain-of-thought reasoning, few-shot examples, or region-specific subprompts. Alternative prompt designs may yield different performance profiles and should be explored in future work.

The present study did not include a direct human comparator on the same image set. A formal comparison between human raters and MLLMs would require a separate, blinded evaluation framework with clearly defined rater populations and standardized assessment procedures. The absence of a human comparator limits the ability to determine whether the observed regional deficits reflect model-specific weaknesses or inherent task difficulty across rater populations.

Model versions represent a snapshot from March 2026 and may differ with subsequent iterations; all models were accessed through web interfaces from Germany. Aesthetic scores and age estimates were exploratory endpoints without external validation.

More broadly, facial image research carries structural concerns regarding data provenance, reuse, and reidentification, because publicly available images may be incorporated into training datasets in nontransparent ways. These do not affect internal validity but reflect a broader constraint of current AI ecosystems.^36^

### Outlook

Future studies should incorporate larger and more diverse datasets, established ordinal wrinkle severity scales (eg, Lemperle and Merz) or photoaging classification systems (eg, Glogau) as reference standards while acknowledging their subjectivity, alongside comparisons with human raters across clinical experience levels.^37-42^

Although our setup avoided spatial positional cues by submitting the 2 images as separate files within the same prompt session without identifying filenames or side-by-side composition, future controlled experiments could further test for residual ordering effects, for instance, by systematically varying the upload sequence or by including merged side-by-side compositions with randomized left/right positioning. Region-specific cropping and systematic prompt variation would additionally help clarify whether the observed behaviors reflect genuine visual reasoning or reliance on superficial image features. Future studies could additionally include decoy image pairs lacking any treatment effect to evaluate the rate at which models continue to predict improvement in the absence of intervention. A blinded single-image grading procedure, in which raters would independently grade each image for wrinkle presence without knowledge of treatment state, could further strengthen the reference standard in future work.

Direct benchmarking against human raters of varying clinical experience on the same image set would help disentangle model-specific limitations from inherent task difficulty. Fine-tuned models trained on expert-annotated aesthetic outcome data represent a promising direction toward clinically viable AI-assisted assessment.^43^

Additionally, future evaluations could explore whether model performance varies with treatment intensity, for instance, by comparing cases of subtle, natural-appearing outcomes with cases of more pronounced muscle inactivity, to assess whether models can distinguish between degrees of aesthetic effect rather than merely detecting treatment presence.

## CONCLUSIONS

MLLMs recognize the format of aesthetic change but not its clinical nuance. Across our 4 contemporary models, perfect treatment-state recognition coexisted with inconsistent region-specific assessment, systematic overestimation, and a clear dissociation between inter-run reliability and classification accuracy. Bridging this gap requires more than improved accuracy. A coordinated research agenda of domain-specific fine-tuning, expert-rater benchmarking on shared image sets, and integration with structured aesthetic outcome frameworks is needed, supported by governance of training-data provenance and clear safeguards against premature deployment of general-purpose models in clinical or research contexts.

## Supplemental Material

This article contains supplemental material located online at www.asjopenforum.com.

## Supplementary Material

ojag112_Supplementary_Data
