# Supplementary material for: Evaluation of Aesthetic Outcomes Following Botulinum Toxin Treatment Using Multimodal Large Language Models: A Paired Before-and-After Analysis
Source: Aesthet Surg J Open Forum. 2026 Jun 13;8:ojag112. doi: 10.1093/asjof/ojag112 (PMC13332406; doi:10.1093/asjof/ojag112)
Supplement: ojag112_Supplementary_Data [file ojag112_supplementary_data.docx]

# Supplementary Material 1: Standardized Evaluation Prompt

You are provided with two facial photographs of the same individual: one taken before and one taken after botulinum toxin treatment of the face. The two images are uploaded as separate files; determine the treatment state from visual content only.

Evaluate the image pair and provide the following outputs. Do not include any additional text, explanation, or commentary.

1. before_after: State which filename corresponds to the pre-treatment image and which corresponds to the post-treatment image.

2. forehead_improvement: Has visible improvement occurred in the forehead region between pre- and post-treatment? (0 = no improvement, 1 = improvement)

3. glabella_improvement: Has visible improvement occurred in the glabella region between pre- and post-treatment? (0 = no improvement, 1 = improvement)

4. periorbital_improvement: Has visible improvement occurred in the periorbital region (crow's feet) between pre- and post-treatment? (0 = no improvement, 1 = improvement)

5. aesthetic_change_score: Rate the overall visible aesthetic improvement between the two images. (integer, 0 to 10; 0 = no visible improvement, 10 = maximal visible improvement)

6. number_of_improved_regions: How many of the three regions (forehead, glabella, periorbital) show visible improvement? (integer, 0 to 3)

7. estimated_age_before: Estimated apparent age of the individual in the pre-treatment image. (integer)

8. estimated_age_after: Estimated apparent age of the individual in the post-treatment image. (integer)

Output format:

before_after: [filename] = BEFORE, [filename] = AFTER

forehead_improvement: [0 or 1]

glabella_improvement: [0 or 1]

periorbital_improvement: [0 or 1]

aesthetic_change_score: [0–10]

number_of_improved_regions: [0–3]

estimated_age_before: [integer]

estimated_age_after: [integer]
